# Synthesis, Spectral and Antimicrobial Studies of Bis(cyclopentadienyl)titanium(IV) Derivatives with Schiff Bases Derived from 2-Amino-5-phenyl-1,3,4-thiadiazole

**DOI:** 10.1155/BCA.2005.289

**Published:** 2005

**Authors:** A. K. Srivastava, O. P. Pandey, S. K. Sengupta

**Affiliations:** Department of Chemistry DDU Gorakhpur University Gorakhpur 273009 India

## Abstract

The reactions of bis(cyclopentadienyl)titanium(IV) dichloride with Schiff bases derived by condensing 2- amino-5-phenyl-1,3,4-thiadiazole with benzaldehyde (SPT), 4-nitrobenzaldehyde (SNT), 4-methoxybenzaldehyde (SMT), 2-hydroxybenzaldehyde (SSTH) or 2-hydroxyacetophenone (SATH) have been studied in refluxing tetrahydrofuran and complexes of types [Cp_2_TiCl(SB)]Cl (SB= SPT, SNT or SMT) and [Cp_2_Ti(SB')]Cl (SB'H= SSTH or SATH) have been isolated. Tentative structural conclusions are drawn for these reaction products based upon elemental analyses, electrical conductance, magnetic moment and spectral (UV-vis, IR and ^1^H NMR) data. Studies were conducted to assess the growth-inhibiting potential of the complexes synthesized, and the ligands, against various fungal and bacterial strains.


### Synthesis, Spectral and Antimicrobial Studies of Bis(cyclopentadienyl)titanium(IV) Derivatives with Schiff Bases Derived from 2-Amino-5-phenyl-1,3,4-thiadiazole

A.K. Srivastava, O.P. Pandey and S.K. Sengupta*

*Department of Chemistry, DDU Gorakhpur University, Gorakhpur- 273009, India*
*E-mail: sengupta2002@yahoo.co.in*

### ABSTRACT

The reactions of bis(cyclopentadienyl)titanium(IV) dichloride with Schiff bases derived by condensing 2-amino-5-phenyl-1,3,4-thiadiazole with benzaldehyde (SPT), 4-nitrobenzaldehyde (SNT), 4-methoxybenzaldehyde (SMT), 2-hydroxybenzaldehyde (SSTH) or 2-hydroxyacetophenone (SATH) have been studied in refluxing tetrahydrofuran and complexes of types $[Cp_2TiCl(SB)]Cl$ (SB= SPT, SNT or SMT) and $[Cp_2Ti(SB')]Cl$ (SB'H= SSTH or SATH) have been isolated. Tentative structural conclusions are drawn for these reaction products based upon elemental analyses, electrical conductance, magnetic moment and spectral (UV-vis, IR and $^1$H NMR) data. Studies were conducted to assess the growth-inhibiting potential of the complexes synthesized, and the ligands, against various fungal and bacterial strains.

### INTRODUCTION

One aspect of the use of coordination compounds of transition metals is their application as biologically active substances /1/. The attention of investigators has focused increasingly on bio-coordination compounds, which can be used as biologically active preparations in medicine and agriculture. The complexes prepared for use in agriculture are, as a rule, more effective at lower concentrations than the metal ions and organic molecules, which enter into their composition. The anticancer and antiviral activities of metal complexes have also been studied. It has also been observed that a small structural change in the ligand may lead to the enhanced activity of the metal complexes. Among the various classes of biologically active coordination compounds, complexes with thiadiazoles as ligands have attracted attention.

Thiadiazole ring is reported /2-5/ to display fungicidal property by virtue of –N=C–S–linkage, which is a possible toxophore in many pesticides. 2,5–Disbstituted 1,3,4–thiadiazole moieties have been found to possess herbicidal, radioprotective, diuretic and bacteriostatic properties. Acetamide derivatives of 2–(benzoyl aminomethyl)–1,3,4-thiadiazole have been found to possess antiarrhythmic, antimetastatic, psychoneurosis, schistosomicidal, fungicidal, herbicidal and pesticidal activities. There are a few reports on transition metal complexes of Schiff bases derived from 2–amino–5–aryl–1,3,4–thiadiazoles. Some transition metal complexes of copper(II), nickel(II), iron(II), cobalt(II), zinc(II), cadmium(II) and silver(I) with 2–amino–5-phenyl-1,3,4– thiadiazole have been prepared and characterized /6,7/. Bismuth(III) and antimony(III) complexes with 2–amino–5–methyl-1,3,4 thiadiazole /8/, the cobalt (II) nickel(II) and copper(II) complexes of 2–amino–5–methyl and 2,5–dimethyl–1,3,4–thiadiazole and ruthenium, rhodium, iridium complexes of 5–amino–1,3,4– thiadiazole–2-thiol have also been reported /9/. The synthesis, characterization and biological activity of cobalt(II), nickel(II) copper(II) and zinc(II) complexes with mercapto thiadiazoles have been reported by Mishra *et al.* /10/. Nath *et al.* reported /11/ the synthesis, spectral, thermal and biological studies of adducts of organotin(IV) halides with Schiff bases derived from 2–amino–5–(*o*–methoxyphenyl)–1,3,4-thiadiazoles. Thus, in view of the versatile chelating ability of thiadiazoles, it has been considered of interest to study the reactions of bis(cyclopentadienyl)titanium(IV) dichloride with substituted thiadiazoles. The structures of the ligands are shown below ( I ).

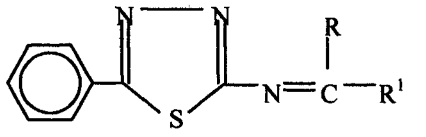

( I )

where R=H, R'= $C_6H_5$ (SPT); R=H, R'= 4-$NO_2.C_6H_4$ (SNT); R=H, R'= 4-$OCH_3$ (SMT); R=H, R'= 2-$OH.C_6H_4$ (SSTH); R= $CH_3$, R= 2-$OH.C_6H_4$ (SATH).

### EXPERIMENTAL

All the reactions were carried out under strictly anhydrous conditions. Tetrahydrofuran (Merck) was dried by sodium wire overnight and then refluxed until it gave a blue colour with benzophenone. The ligands, Schiff bases, were prepared as mentioned in the literature /11/. Bis(cyclopentadienyl)titanium(IV) dichloride was prepared /12/ by heating CpNa with $TiCl_4$ in a $N_2$ atmosphere. Elemental analyses and physical measurements were made as noted earlier /13/.

### Synthesis of Complexes

***(a) Reaction of bis(cyclopentadienyl)titanium(IV) chloride with Schiff base derived from 2–amino–5–phenyl–1,3,4–thiadiazole and benzaldehyde (SPT) (molar ratio 1:1)***

Schiff base (SPT) (0.02 mol) was added to a refluxing solution of bis(cyclopentadienyl)titanium(IV) dichloride (0.02 mol) in anhydrous tetrahydrofuran (40 $cm^3$). The reaction mixture was refluxed for 60 hrs. The brown coloured complex so obtained was filtered, washed with anhydrous tetrahydrofuran and dried in *vacuo*. The complex was identified to be $[(C_5H_5)_2TiCl(C_{15}H_{11}N_3S)]Cl$. Yield : 58%

The same procedure was adopted for the synthesis of other Schiff bases (derived by the condensation of 2–amino–5–phenyl–1,3,4–thiadiazole and 4–nitrobenzaldehyde (SNT) or 4–methoxy benzaldehyde (SMT)) derivatives of bis(cyclopentadienyl)titanium(IV). For the sake of brevity, these reactions are summarized in Table 1. The analytical data of the products are given in Table 1.

**Table 1**

Reactions of bis(cyclopentadienyl)titanium(IV) dichloride with Schiff bases derived from 2–amino–5–phenyl–1,3,4–thiadiazoles

| Reactants | Stirring/ refluxing time(hrs) | Solvent | Product, colour Yield (%), Decomp. Temp (°C) | Calcd. (Found) % | | | | |
|---|---|---|---|---|---|---|---|---|
| | | | | C | H | N | S | Ti |
| $Cp_2TiCl_2$ | 60 | THF | $[Cp_2TiCl(SPT)]Cl$ | 58.39 | 4.11 | 8.17 | 6.23 | 9.30 |
| +SPT | | | Yellow, 58, 120 | (58.10) | (4.00) | (8.02) | (6.05) | (9.12) |
| $Cp_2TiCl_2$ | 55 | THF | $[Cp_2TiCl(SNT)]Cl$ | 54.08 | 3.62 | 10.09 | 5.78 | 8.62 |
| +SNT | | | Dark yellow, 55, 150 | (53.90) | (3.42) | (9.98) | (5.66) | (8.58) |
| $Cp_2TiCl_2$ | 60 | THF | $[Cp_2TiCl(SMT)]Cl$ | 57.38 | 4.26 | 7.72 | 5.89 | 8.80 |
| +SMT | | | Brick red, 60, 122 | (57.04) | (4.18) | (7.66) | (5.72) | (8.72) |
| $Cp_2TiCl_2$ | 45 | $CH_2Cl_2$ | $[Cp_2Ti(SST)]Cl$ | 58.90 | 3.95 | 8.24 | 6.29 | 9.40 |
| +SSTH | | | Yellow, 72, 110 | (58.73) | (3.78) | (8.15) | (6.16) | (9.34) |
| $Cp_2TiCl_2$ | 45 | $CH_2Cl_2$ | $[Cp_2Ti(SAT)]Cl$ | 61.49 | 4.37 | 8.27 | 6.31 | 9.42 |
| +SATH | | | Yellow, 70, 142 | (61.20) | (4.18) | (8.16) | (6.15) | (9.30) |

where,

SPT = Schiff base derived form 2–amino–5–phenyl–1,3,4–thiadiazole and benzaldehyde
SNT = Schiff base derived form 2–amino–5–phenyl–1,3,4–thiadiazole and 4–nitrobenzaldehyde
SMT = Schiff base derived form 2–amino–5–phenyl–1,3,4–thiadiazole and 4–methoxybenzaldehyde
SSTH = Schiff base derived form 2–amino–5–phenyl–1,3,4–thiadiazole and salicylaldehyde
SATH = Schiff base derived form 2–amino–5–phenyl–1,3,4–thiadiazole and 2–hydroxy acetophenone

***(b) Reaction of bis(cyclopentadienyl)titanium(IV) dichloride with Schiff base derived from 2–amino–5–phenyl–1,3,4–thiadiazole and salicylaldehyde (SSTH) (molar ratio 1:1)***

To the mixture of bis(cyclopentadienyl)titanium(IV) dichloride (0.01 mol) and Schiff base (SSTH) (0.01 mol) was added anhydrous dichloromethane (60 $cm^3$) followed by 45 hours reflux. The volume of the solution was reduced to about 10 $cm^3$. Dry petroleum ether (b.p. 60–80°, 20 $cm^3$) was added to it and the solution was allowed to stand for one hour, which resulted in yellow crystals of the product. The complex, thus obtained, was filtered and dried in oven at 80°C. The complex was found to be $[(C_5H_5)_2$-$Ti(C_{15}H_{10}N_3SO)]Cl$. Yield 72%.

The same procedure was adopted for the synthesis of bis(cyclopentadienyl)titanium(IV) derivatives with Schiff bases derived from 2–amino–5–phenyl–1,3,4–thiadiazole and *o*–hydroxy acetophenone (SATH). For the sake of brevity, such reactions are summarized in Table 1. The analytical data of the products are given in Table 1.

### RESULTS AND DISCUSSION

A systematic study of the reactions of bis(cyclopentadienyl)titanium(IV) dichloride with neutral (SB) and monobasic (SB'H) Schiff bases in (1:1) molar ratio have been carried out. The reactions can be represented by the following equations.

$$Cp_2TiCl_2 + SB \xrightarrow[\text{reflux}]{\text{THF}} [Cp_2TiClSB]Cl$$

$$Cp_2TiCl_2 + SB'H \xrightarrow[\text{reflux}]{CH_2Cl_2} [Cp_2TiClSB']Cl$$

where SB= SPT, SNT or SMT; $SB^1H$= SSTH and SATH.

The elemental analyses (Table 1) indicate a 1:1 metal to ligand stoichiometry for all these complexes. The compounds are yellow to dark brown in colour, non-volatile and moisture sensitive. The solid products obtained are thermally stable but decompose on heating. The complexes of the type $[(C_5H_5)_2TiCl(SB)]Cl$ are soluble in dimethylformamide and dimethyl- suplhoxide, while the complexes of the type $[(C_5H_5)_2Ti(SB')]Cl$ are sparingly soluble in hot methanol, ethanol and soluble in dimethylformamide and dimethylsulphoxide. Electrical conductance measurements of the complexes in dimethylformamide indicate their 1:1 electrolytic nature. Magnetic susceptibility values of these complexes at room temperature show their diamagnetic behavior.

### Electronic spectra

The electronic spectra of all Schiff base derivatives in nujol show bands in the regions 23,500–24,200, 30,000 and 34,800 $cm^{-1}$. The first band may be assigned /14/ to the charge-transfer band and its in accordance with their $(n-1)d^0ns^0$ electronic configuration. The second and third bands (also appear in ligands) are assigned to $\pi \rightarrow \pi^*$ transitions of the azomethine linkages.

### Infrared spectra

The significant infrared spectral bands of the complexes are given in Table 2. A comparison of the characteristic infrared absorption bands of Schiff bases with those of the corresponding titanium(IV) derivatives reveals the following important features.

(a) The infrared spectra of the ligands show bands at *ca.* 1650–1635 and 1580–1565 $cm^{-1}$ which may be assigned /2/ to be ν(C=N) (azomethine group) and ν(C=N) (ring), respectively. The first band is shifted towards the lower frequency region (20–30 $cm^{-1}$) in the titanium(IV) complexes indicating the coordination of the azomethine nitrogen atom of the Schiff bases. However, the second band νC=N (ring) is found to split into two; one almost located at the original position indicating non–coordinated νC=N, and the other shifted to lower frequency at 1550–1540 $cm^{-1}$, arising due to coordinated νC=N mode. The splitting of the νC=N (ring) absorption band suggests that only one of the ring nitrogen is involved in coordination and other is free and non-coordinated. This is further supported by a new band at *ca.* 450–440 $cm^{-1}$ assignable /15/ to ν(Ti–N) in the complexes.

(b) The ν(C–S) band in the ligands occurs /16/ at *ca.* 650 $cm^{-1}$, which remains unaltered in the spectra of the complexes indicating the non–coordination of sulphur atom of the thiadiazole ring.

(c) The infrared spectra of the ligands containing –OH group (SSTH, SATH) show strong band at *ca.* 3400 $cm^{-1}$ due to ν(O–H). However, this band disappears in the spectra of their corresponding complexes indicating the coordination of phenolic oxygen to metal. The ν(Ti–O) band appears at ~500–480 $cm^{-1}$.

(d) Absorption bands occurring at ~3000 $cm^{-1}$ for ν(C–H), ~1430 $cm^{-1}$ for ν(C–C), ~ 1020 and 810 $cm^{-1}$ for δ(C–H) in the complexes indicate the presence of the cyclopentadienyl ring. All these bands are similar to those reported /17/ for bis(cyclopentadienyl)titanium(IV).

**Table 2**

IR spectral data ($cm^{-1}$) of bis(cyclopentadienyl)titanium(IV) complexes with Schiff bases

| Compound | ν(C=N) | ν(C=N) (ring) | ν(C-S) | ν(Ti-N) | ν(Ti-O) | $C_5H_5$ |
|---|---|---|---|---|---|---|
| SPT | 1650 s | 1580 m | 650 m | _ | _ | _ |
| [$Cp_2$TiCl(SPT)]Cl | 1625 s | 1580 m<br>1550 m | 650 m | 450 m | _ | 3000 s, 1430 m,<br>1020 w, 810 w |
| SNT | 1635 s | 1565 m | 640 m | _ | _ | _ |
| [$Cp_2$TiCl(SNT)]Cl | 1610 s | 1565 m<br>1540 m | 645 m | 440 m | _ | 2990 w, 1420 m,<br>1110 w, 800 w |
| SMT | 1650 s | 1580 m | 650 m | _ | _ | _ |
| [$Cp_2$TiCl(SMT)]Cl | 1630 s | 1585 m<br>1550 m | 655 m | 445 m | _ | 3110 s, 1425 m,<br>1115 w, 810 w |
| SSTH | 1640 s | 1575 m | 645 m | _ | _ | _ |
| [$Cp_2$Ti(SST)]Cl | 1620 s | 1570 m<br>1545 m | 640 m | 442 m | 500 m | 3000 s, 1428 m,<br>1115 w, 800 w |
| SATH | 1650 s | 1565 m | 645 m | _ | _ | _ |
| [$Cp_2$Ti(SAT)]Cl | 1620 s | 1568 m<br>1540 m | 645 m | 440 m | 480 m | 3000 s, 1420 m,<br>1120 w, 805 w |

### $^{1}H$ NMR spectra

The different proton magnetic resonance signals (recorded in DMSO-$d_6$) are given in Table 3. The intensities of all the resonance lines were determined by planimetric integration. Comparing the spectra of ligands and their corresponding complexes, one can derive the following conclusions.

(a) A signal in all Schiff base derivatives at δ ~ 6.5–6.8 may be assigned to the protons of the cyclopentadienyl ring. The appearance of a single, sharp cyclopentadienyl resonance is attributed /18/ to the rapid rotation of the cyclopentadienyl ring around the metal ring axis.

(b) All the ligands show signal at δ 7.1-7.5 integrating for aromatic group protons. This signal shows slight shift upon co-ordination.

(c) The signal observed at δ 9.4–9.8 in the spectra of ligands, derived from salicylaldehyde and *o*–hydroxyacetophene may be assigned to the phenolic protons. This signal disappears in the spectra of their corresponding complexes indicating the coordination through deprotonation of phenolic oxygen atoms.

(d) The proton signal due to the CH=N group appears at ~ δ 8.3 in the ligands. In the complexes, this signal shifts downfield which is probably due to the donation of the lone pair of electrons by the azomethine nitrogen to the titanium atom.

**Table 3**

$^{1}H$ NMR spectral data (δ scale, ppm) of bis(cyclopentadienyl)titanium(IV) complexes with Schiff base

| Complex | $\eta^5$–$C_5H_5$ | Phenyl ring | –CH = N | –$CH_3$ |
|---|---|---|---|---|
| SPT | _ | 7.20–7.48(m) | 8.32(s) | _ |
| [$(C_5H_5)_2$TiCl(SPT)]Cl | 6.50(s) | 7.25 – 7.52(m) | 8.45(s) | - |
| SNT | _ | 7.22–7.55(m) | 8.30(s) | _ |
| [$(C_5H_5)_2$TiCl(SNT)]Cl | 6.60(s) | 7.28 – 7.60(m) | 8.48(s) | - |
| SMT | _ | 7.10–7.50(m) | 8.38(s) | 2.26(s) |
| [$(C_5H_5)_2$TiCl(SMT)]Cl | 6.80(s) | 7.15 – 7.55(m) | 8.50(s) | 2.30(s) |
| SSTH | _ | 7.22–7.58(m) | 8.32(s) | _ |
| [$(C_5H_5)_2$Ti(SST)]Cl | 6.70(s) | 7.30 – 7.62(m) | 8.42(s) | - |
| SATH | _ | 7.18–7.52(m) | _ | 2.08(s) |
| [$(C_5H_5)_2$Ti(SAT)]Cl | 6.65(s) | 7.25 – 7.58(m) | - | 2.10(s) |

Thus, it is obvious from the above discussion that the Schiff bases (SPT, SNT, SMT) act as neutral, bidentate ligands coordinating through azomethine nitrogen and one of the nitrogen of the thiadiazole ring, whereas the Schiff bases SSTH, SATH act as monobasic, tridentate chelating agents coordinating through azomethine nitrogen, one of the nitrogens of the thiadiazole ring and deprotonated phenolic oxygen atom. Therefore, on the basis of above spectral features the following structures may be proposed for $[Cp_2TiCl.SB]Cl$ (II) and $[Cp_2Ti.SB']Cl$ (III) complexes.

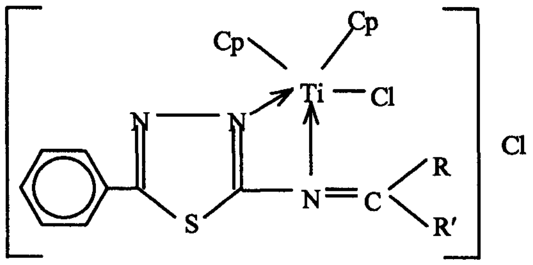

(II)

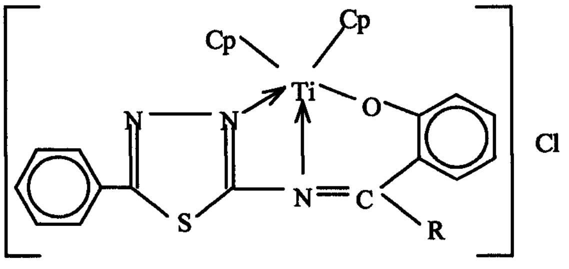

(III)

### Antimicrobial Studies

### *Antifungal activity*

**Table 4**

Antifungal activity of Schiff bases and their corresponding bis(cyclopentadienyl)titanium(IV) complexes

| Compound | Average % inhibition after 96 h | | | | | | | | |
|---|---|---|---|---|---|---|---|---|---|
| | *A. niger* | | | *A. alternata* | | | *H. oryzae* | | |
| | 1000 | 100 | 10 | 1000 | 100 | 10 | 1000 | 100 | 10 |
| SPT | 53.8 | 48.2 | 36.8 | 52.8 | 42.7 | 33.6 | 52.6 | 43.5 | 31.6 |
| $[Cp_2TiCl(SPT)]Cl$ | 60.2 | 52.8 | 42.0 | 61.8 | 50.6 | 44.2 | 60.5 | 50.8 | 41.0 |
| SNT | 73.2 | 62.5 | 58.2 | 70.8 | 60.1 | 55.3 | 68.2 | 58.7 | 50.2 |
| $[Cp_2TiCl(SNT)]Cl$ | 80.1 | 72.6 | 60.2 | 76.2 | 70.8 | 58.6 | 70.0 | 62.6 | 52.8 |
| SMT | 80.2 | 70.5 | 60.4 | 72.6 | 65.6 | 58.2 | 70.2 | 61.8 | 55.2 |
| $[Cp_2TiCl(SMT)]Cl$ | 88.2 | 78.4 | 65.8 | 80.4 | 72.8 | 62.3 | 78.8 | 70.2 | 60.8 |
| SSTH | 69.6 | 60.2 | 51.3 | 66.8 | 53.2 | 48.0 | 61.2 | 50.5 | 42.8 |
| $[Cp_2Ti(SST)]Cl$ | 76.2 | 69.8 | 58.0 | 70.1 | 60.2 | 52.3 | 68.8 | 52.0 | 48.2 |
| SATH | 60.1 | 57.2 | 50.0 | 60.0 | 54.2 | 43.6 | 54.2 | 42.8 | 40.4 |
| $[Cp_2Ti(SAT)]Cl$ | 70.2 | 62.1 | 53.2 | 66.1 | 60.5 | 50.2 | 60.2 | 50.8 | 46.3 |

Conc. used in ppm

The fungicidal activity of the ligands and the complexes were evaluated in DMF against *Aspergillus niger, Aspergillus alternata* and *Helminthosporium oryzae* by the agar plate technique /19/ at 1000, 100 and 10 ppm concentrations with triplicate determinations in each case. The average percentage inhibition was calculated using the expression: inhibition (%)= 100 (C-T)/C, where C and T are the diameters of the fungus colony in control and test plates, respectively. The recorded results (Table 4) lead to the following conclusions:

(a) The compounds show significant toxicity at 1000 ppm conc. Against all species of fungi. However, the complexes are more active than ligands, which may be owing to the chelation and the presence of sulphur atom.

(b) The activity decreases on dilution.

(c) The best activity was noted for complex with ligand derived from 4-methoxybenzaldehyde. This indicates that the presence of methoxy group imparts the fungicidal power in these series of compounds.

(d) The ligands and complexes are more active against *A. niger* than *A. alternata* and *H. oryzae.*

The variation in the effectiveness of different biocidal agents against different organisms, as suggested by Lawrence *et al.* /20/ depends either on the impermeability of the cells or differences in ribosomes of antimicrobial agent.

***Antibacterial activity***

The antibacterial activity of the complexes together with parent ligands has been screened against Gram-positive *Bacillus subtilis* and Gram-negative *Escherichia coli* by the paper disk plate method /21/ at 1000 ppm conc. The inhibition zone (mm) around each disk was measured after 24 h and the results of these studies are listed in Table 5. It is clear from the screening data that complexes are slightly more toxic than the parent ligands.

**Table 5**

Antibacterial activity of Schiff bases and their corresponding bis(cyclopentadienyl)titanium(IV) complexes

| Compound | *Diameter of inhibition zone (mm)* | |
|---|---|---|
| | *B. Subtilis* | *E. Coli* |
| SPT | 10 | 12 |
| $[Cp_2TiCl(SPT)]Cl$ | 14 | 15 |
| SNT | 12 | 14 |
| $[Cp_2TiCl(SNT)]Cl$ | 14 | 15 |
| SMT | 16 | 16 |
| $[Cp_2TiCl(SMT)]Cl$ | 20 | 18 |
| SSTH | 15 | 16 |
| $[Cp_2Ti(SST)]Cl$ | 16 | 17 |
| SATH | 14 | 15 |
| $[Cp_2Ti(SAT)]Cl$ | 16 | 17 |
| Ampicillin | 35 | 35 |

### ACKNOWLEDGEMENT

One of the authors (SKS) thanks the University Grants Commission, New Delhi for financial assistance.

### REFERENCES

1. G. Wilkinson, R.D. Gillard and J.A. McCleverty (Eds), *Comprehensive Coordination Chemistry*, Vol. 6(1987).
2. S. Srivastava, V. Srivastava, K. Chaturvedi, O.P. Pandey and S.K. Sengupta, *Thermochim. Acta*, **240**, 101(1994) and references therein.
3. Hao Xin, *Hecheng Huaxue*, **8**, 252(2000); *Chem. Abstr.*, **134**, 17345(2001).
4. A. Louria, *J. Heterocyclic Chem.*, **37**, 747(2000).
5. Z.A. Hozien, *J. Heterocyclic Chem.*, **37**, 943(2000).
6. N.B. Singh and J. Singh, *J. Inorg. Nucl. Chem.*, **41**, 1384(1979).
7. A.C. Fabretti, G.C. Franchini and G. Peyronel, *Spectrochim. Acta*, **36A**, 689(1980).
8. A.C. Fabretti, G.C. Franchini and G. Peyronel, *Inorg. Chim. Acta*, **42**, 217(1980).
9. K.N. Johri and B.S. Arora, *Thermochim. Acta*, **54,** 237(1982).
10. S. Mishra, B.L. Dubey and S.C. Bahel, *Synth. React. Inorg. Met.-Org. Chem.*, **21**, 637(1991).
11. Mala Nath, S. Goyal, G. Eng and D. Whalen, *Bull. Chem. Soc. Jpn*, **69**, 605(1996).
12. G. Wilkinson and J.M. Birmingham, *J. Am. Chem. Soc.*, **76**, 4281(1954).
13. O.P. Pandey, S.K. Sengupta, M.K. Mishra and C.M. Tripathi, *Bioinorg. Chem. Appl.*, **1**, 35(2003).
14. R. Rai, K.D. Mishra, O.P. Pandey and S.K. Sengupta, *Polyhedron*, **11**, 123(1992).
15. A. Mala, A.K. Srivastava, O.P. Pandey and S.K. Sengupta, *Transition Met. Chem.*, **25**, 613(2000).
16. K. Nakamoto, *Infrared and Raman spectra of inorganic and coordination compounds*, Part B, John Wiley & Sons, Inc., New York, 1997.
17. E. Hey-Hawkins, *Chem. Rev.*, **94**, 1661(1994).
18. S.K. Sengupta, O.P. Pandey and S.K. Srivastava, *Indian J. Chem.*, **38A**, 1066(1999).
19. V.K. Sharma, O.P. Pandey and S.K. Sengupta, *J Inorg. Biochem.*, **34**, 253(1988).
20. P.G. Lawrence, P.L. Harold and O.G. Francis, *Antibiot. Chemother.*, 1597(1980).
21. S.P. Shahi, *Ph.D. Thesis*, DDU Gorakhpur University (India), 1998.

